# Suicidal Ideation, Psychological Distress and Child-To-Parent Violence: A Gender Analysis

**DOI:** 10.3389/fpsyg.2020.575388

**Published:** 2020-12-17

**Authors:** Belén Martínez-Ferrer, Ana Romero-Abrio, Celeste León-Moreno, María Elena Villarreal-González, Daniel Musitu-Ferrer

**Affiliations:** ^1^Education and Social Psychology Department, Pablo de Olavide University, Seville, Spain; ^2^Faculty of Education, International University of La Rioja, La Rioja, Spain; ^3^Faculty of Psychology, Autonomous University of Nuevo Leon, Monterrey, Mexico

**Keywords:** child-to-parent violence, adolescence, suicidal ideation, psychological distress, self-concept

## Abstract

Child-to-parent violence (CPV) is a growing public health problem with consequences for perpetrators and families. Most research has focused on individual and family risk factors. However, little is known about its links with individual outcomes. The aim of this study was to analyze the relationships between CPV and psychological distress, suicidal ideation, and self-concept in school-aged adolescents, taking into account the gender perspective. A study was conducted with a sample of 8,115 adolescents, aged between 11 and 16 years (*M* = 13.34; *SD* = 1.04) from the State of Nuevo León, Mexico. A MANOVA 3 × 2 was performed to analyze the data. The results revealed that adolescents involved in CPV showed higher levels of psychological distress and suicidal ideation and lower levels of family and social self-concept. It was also observed that girls with higher levels of CPV scored the lowest levels of psychological distress and suicidal ideation, as well as the lowest levels of family self-concept. The findings highlight that adolescents and especially girls involved in CPV also report internal maladjustment outcomes. Finally, the results and their implications for research and intervention with adolescents involved in CPV are discussed.

## Introduction

Child-to-parent violence (CPV) is defined as repeated behaviors of physical, psychological, or economic violence directed at parents (Pereira et al., 2017; Arias-Rivera and García, 2020). The increase in the prevalence of CPV in the last decade (Condry and Miles, 2014) has fueled great social concern (Holt, 2016). In studies conducted in different countries, the prevalence rates of CPV varied: in the US, rates ranged from 14 to 20% in physical CPV and from 34 to 64% in verbal and psychological CPV (Pagani et al., 2004, 2009; Lyons et al., 2015). In Spain, previous studies made with community samples have indicated different values: prevalence ranged from 4.6 to 21% in physical CPV (Calvete et al., 2011; Ibabe and Jaureguizar, 2011); and between 34 and 93% in psychological CPV (Pagani et al., 2004, 2009; Calvete et al., 2017). In a study carried out in Mexico by Calvete and Veytia (2018), a prevalence rate between 72 and 87.2% was obtained in psychological CPV and in the physical CPV dimension the rate was similar to that of Spain. In terms of the gender of the aggressors, it has been found that, on average, CPV is more common in boys than in girls (Aroca-Montolío et al., 2014; Martínez-Ferrer et al., 2018). Regarding the type of CPV (physical or psychological), in previous works different results have been observed, depending on the sample used (Calvete and Veytia, 2018; Del Hoyo-Bilbao et al., 2020). Thus, some studies carried out with clinical or judicial samples have pointed out that boys use physical CPV more often than girls (Boxer et al., 2009; Routt and Anderson, 2011). However, other works made with community samples have observed that psychological CPV is more frequently used in girls (Ulman and Straus, 2003; Ibabe and Jaureguizar, 2011; Calvete et al., 2013; Calvete and Orue, 2016; Rosado et al., 2017). Nevertheless, in more recent studies, no gender differences were found in psychological CPV (Ibabe et al., 2013).

Involvement in CPV is the result of the complex interplay between individuals and their broader social environment. Based on the ecological model by Bronfenbrenner (1979), the Nested Ecological Model (Dutton, 1985; Cottrell and Monk, 2004) has been used to explain that CPV is the result of the interaction between the individual, family, school, peer group, community, and society contexts. In this regard, it has been found that risk factors at the micro-(e.g., parenting behavior), meso-(e.g., peer influence), exo-(e.g., media influence), macro-(e.g., gender role socialization), and chronosystem (change in family structure) level are likely to influence adolescents’ violence toward their parents (Hong et al., 2012). In numerous studies ontogenetic variables associated with CPV have been analyzed, however, there are certain factors that have received less attention from researchers which we consider important, such as suicidal ideation, psychological distress, and self-concept. In relation to the differences between CPV toward the mother and father, previous studies have found that adolescents engage in greater verbal violence toward the mother, and greater physical violence toward the father (Lyons et al., 2015). In addition, other authors have pointed out that CPV toward the mother is linked with family variables such as physical punishment, while other factors such as impulsivity, substance abuse, or the impossibility of imposing discipline on the children are related to CPV regardless of the parent gender (Del Hoyo-Bilbao et al., 2020). The present study aimed to analyze the relationships between the variables at the individual level and CPV, taking into account the gender of the perpetrator.

According to the General Strain Theory (Agnew, 2006), CPV could represent a maladaptive response to stress or dysfunctional resources to face stress in adolescents (Brezina, 1999). Psychological distress is defined as a state of emotional suffering characterized by symptoms of depression, anxiety, and somatic symptoms as a consequence of stressors and demands that are difficult to cope with in daily life (Mirowsky and Ross, 2002; Drapeau et al., 2012). Adolescents with high levels of psychological distress are likely to be involved in CPV (Kennedy et al., 2010; Gámez-Guadix and Calvete, 2012). Therefore, CPV could be a strategy that may buffer the detrimental effects of high psychological distress (Beckmann, 2019). Defiance of rules and authority and aggression may be a self-protective strategy to face stress and adversity (Ford et al., 2006) and a negative way to try to emotionally overcome negative situations (Siegel, 2013).

Certain protector factors are related to lower involvement in CPV, despite psychological distress. Previous studies have highlighted that adolescents who reported lower levels of CPV have high levels of emotional regulation (Martínez-Ferrer et al., 2018) and empathy (Cottrell and Monk, 2004). In this sense, self-concept is an important resource that is associated with lower levels of psychological distress (Khalaila, 2015; Grubbs and Exline, 2016; Turner et al., 2017) and with low involvement in CPV (Calvete et al., 2011). However, other authors observed no significant differences in self-esteem between CPV offenders and CPV non-offenders (Ibabe et al., 2014; Contreras and Cano, 2015; Loinaz and Sousa, 2020). Nevertheless, these studies provided a general measure of self-esteem or self-concept. Prior studies have reported that self-concept, evaluated from a multi-dimensional perspective, is related to different forms of violence such as peer aggression and intimate partner violence (García et al., 2006; Fuentes et al., 2011). In particular, previous studies concluded that family self-concept protects adolescents from getting involved in peer aggression and bullying (Romero-Abrio et al., 2019), whereas social self-concept is positively related to adolescent involvement in peer and school aggression as perpetrators (Usán and Salavera, 2017; Chacón-Cuberos et al., 2020). However, no study to date has focused on these particular dimensions of self-concept.

Both psychological distress and self-concept are related to suicidal ideation among adolescents (Espinoza-Gómez et al., 2010; Maraš et al., 2011; Ramírez and Oduber, 2015). Furthermore, it has been pointed out that suicidal ideation is linked to adolescent involvement in peer aggression, bullying, cyberbullying, victimization, and intimate peer violence (Garaigordobil, 2011; Buttar et al., 2013; Kowalski et al., 2014; Romero-Abrio et al., 2018; Iranzo et al., 2019). Despite the scarcity of research focusing on suicidal ideation and CPV, prior studies have concluded that CPV is associated with greater suicidal ideation (Clarke et al., 2017). Moreover, a recent study showed that girls involved in CPV were more likely to report psychological distress and suicidal ideation than boys (Armstrong et al., 2018).

### The Present Study

Prior research has analyzed CPV taking into account socio-ecological theoretical frameworks. The present study also takes into account the General Strain Theory (Agnew, 2006) and examines the relationships between CPV and psychological distress, suicidal ideation, and family and social self-concept. Adolescents with high levels of involvement in CPV were expected to report the lowest level of psychological adjustment—high levels of psychological distress, suicidal ideation, and social self-concept, and low levels of family self-concept. It has been consistently reported that CPV is more frequent among boys (Arias-Rivera and García, 2020; Del Hoyo-Bilbao et al., 2020). However, girls tend to show higher levels of psychological distress (Hamilton et al., 2016; Hébert et al., 2016), suicidal ideation (Hinduja and Patchin, 2010; Nock et al., 2013), and family self-esteem (Birndorf et al., 2005; Fuentes et al., 2011; Romero-Abrio et al., 2019) than boys. Therefore, gender was considered in the present study.

Based on the foregoing research, the following hypotheses were proposed:

H1: Adolescents reporting higher levels of CPV were expected to show higher levels of psychological distress and suicidal ideation.

H2: Adolescents with low levels of involvement in CPV were expected to show higher levels of family and social self-concept.

H3: Girls were expected to report higher levels of psychological distress, suicidal ideation, and family self-concept, and lower levels of social self-concept than boys.

H4: Girls highly involved in CPV were expected to report the most adverse maladjustment outcomes, characterized by the highest levels of psychological distress and suicidal ideation and the lowest level of family and social self-concept.

## Materials and Methods

### Participants

Proportional stratified sampling was carried out according to urban and rural educational centers (a total of 984 centers) in the State of Nuevo León (Mexico) (confidence level 90%, alpha 0.05). A total of 8,115 adolescents participated (51.5% boys and 48.5% girls) from 118 centers (62 urban and 56 rural), of which 62.1% studied in urban schools and 37.9% studied in rural schools. The ages ranged from 12–13 years (53.7%) to 14–16 years (46.3%). Data lost by scale or sub-scale, provided they did not exceed 15%, were processed using the multiple linear regression imputation model (Allison, 2000; Fernández-Alonso et al., 2012). Univariate atypical data were detected by exploration of standardized scores (Hair et al., 1999).

### Measures

*Conflict Tactics Scales, CTS2, children to parents version* (Straus and Douglas, 2004) adapted by Gámez-Guadix et al. (2010). This two-factor scale is composed of 6 Likert-type items with four response options (1 = never, 4 = more than 20 times) that assesses violence toward the mother and the father, separately (e.g., “I threaten or have threatened to beat up my parents, but I haven’t”). The scale allows for two factors to be scored (physical violence and verbal violence); and an overall rating of CPV. Cronbach’s alpha in this study were 0.70 for the subscale of violence toward the mother (0.71 and 0.75 for physical and verbal violence, respectively), 0.75 for the subscale of violence toward the father (0.85 and 0.70 for physical and verbal violence, respectively), and 0.71 for the full scale. The CFA using the Maximum Likelihood model presented an acceptable fit to the data [SBχ^2^ = 52.8465, gl = 20, *p* < 0.001, CFI = 0.975, RMSEA = 0.014 (0.010, 0.019)] for the subscale of violence toward the mother; and [SBχ^2^ = 82.0587, *df* = 22, *p* < 0.001, CFI = 0.963, RMSEA = 0.018 (0.014, 0.023)] for the subscale of violence toward the father.

*Psychological Distress Scale* (K10) (Kessler and Mroczek, 1994). It consists of 10 Likert-type items with five response options (1 = never, 5 = always) that assesses depressive and anxiety symptoms (e.g., “How often did you feel so sad that nothing could cheer you up?”). Cronbach’s alpha was 0.90. The CFA showed a good fit to the data [SBχ^2^ = 512.36, *df* = 29, *p* < 0.001, CFI = 0.981, RMSEA = 0.045 (0.042, 0.049)].

*Suicidal Ideation Scale* (Roberts, 1980), adapted by Mariño et al. (1993). It consists of 4 Likert-type items with four response options (1 = 0 days, 4 = 5–7 days) that rates the frequency of suicidal thoughts during the previous week (e.g., “I felt that my family would be better if I were dead”). Cronbach’s alpha was 0.84. The CFA presented a good fit to the data [SBχ^2^ = 1.643, *df* = 1, *p* = 0.199, CFI = 0.991, RMSEA = 0.009 (0.000, 0.032)].

*Form-5 Scale*—AF-5—(García and Musitu, 1999). For the purposes of the present study the social and family self-concept subscales were selected. The family self-concept subscale is composed of 6 items that assesses adolescent self-perception in the family context (e.g., “At home they criticize me a lot” reverse item). The social self-concept subscale consists of 6 items that assesses adolescent self-perception in a social context (e.g., “I make friends easily”). Both subscale responses ranged from 1 = completely disagree to 99 = completely agree. Cronbach’s alpha in this study was 0.77 for the family self-concept and 0.88 for social self-concept. The CFA using the Maximum Likelihood model presented an acceptable fit to the data [SBχ^2^ = 6,892.5998, *df* = 337, *p* < 0.001, CFI = 0.958, RMSEA = 0.050 (0.049, 0.051)].

### Procedure

Researchers from the Autonomous University of Nuevo León (Mexico) in collaboration with the Pablo de Olavide University (Spain) carried out the planning and the research. First, an informative seminar was held with the students to explain the objectives, the scope of the study, and the procedure to be followed. Then, the necessary authorizations were obtained from school administrators and participating families were requested to give active parental consent for their child to participate in the study. The battery of instruments was administered voluntarily, anonymously, and supervised in two different sessions of approximately 25 min during school hours with a 15 min rest period between sessions. The questionnaires were answered individually on paper, administered in groups, and supervised by a group of previously trained researchers. Participation was voluntary and anonymous, with a rejection rate of 0.21%. It is important to underline that the study fulfilled the ethical values required in research with human beings, respecting the fundamental principles included in the Helsinki Declaration (World Medical Association, 2013): informed consent and the right to information, protection of personal data and guarantees of confidentiality, non-discrimination, gratuity, and the possibility of withdrawing from the study at any stage.

#### Ethics

The studies involving human participants were reviewed and approved by Institutional Review Board of the Faculty of Psychology of Autonomous University of Nuevo Leon.

### Data Analysis

First, in order to obtain an optimal number of clusters, a two-stage cluster analysis was performed using the two dimensions of CPV (physical violence and verbal violence) toward the mother and the father. Three clusters were obtained with a good fit: low, moderate, and high CPV. Next, the k-means cluster analysis was performed. Finally, a multivariate factorial design was carried out (MANOVA 3 × 2) with CPV (high, moderate, and low) and gender (boy and girl) as fixed factors, and as dependent variables, psychological distress, suicidal ideation, and self-concept (family self-concept and social self-concept), in order to analyze possible interaction effects. SPSS software (version 25) was used.

## Results

### Descriptive Analysis

Table 1 shows the distribution of adolescents according to CPV and gender, psychological distress, suicidal ideation, and self-concept. The percentage of boys and girls was similar in all the variables.

**TABLE 1:** Sociodemographic variables.

		CPV			Total
		Low	Moderate	High	
**Gender**					
Boys	*N*	3,381	680	116	4,177
	%	80.9	16.3	2.8	100
Girls	*N*	2,797	924	217	3,938
	%	71.0	23.5	5.5	100
Total	*N*	6,178	1,604	333	8,115
	%	76.1	19.8	4.1	100

### Multivariate Analysis

A MANOVA was carried out and significant differences were obtained in the main effects of CPV [Λ = 0.895, *F*(8, 16,210) = 159.598, *p* < 0.001, η^2^ = 0.073], and gender [Λ = 0.977, *F*(4, 8,105) = 48.030, *p* < 0.001, η^2^ = 0.023] (see Table 2). The effect size of η^2^ is between moderate and low. Moreover, a statistically significant interaction for CPV and gender [Λ = 0.992, *F*(8, 16,210) = 7.735, *p* < 0.001, η^2^ = 0.004] was obtained.

**TABLE 2:** MANOVA of suicidal ideation, psychological distress, family self-concept, and social self-concept.

Variables						
	Λ	*F*	gl_*entre*_	gl_*error*_	*p*	η^2^
(A) CPV^a^	0.859	159.598	8	16,210	< 0.001***	0.073
(B) Gender^b^	0.977	48.030	4	8,105	< 0.001***	0.023
A × B	0.992	7.735	8	16,210	< 0.001***	0.004

^a^a_1_, low CPV, a_2_, moderate CPV, a_3_, high CPV. ^b^b_1_, boys, b_2_, girls. ***p < 0.001.

Regarding CPV, the ANOVA results found significant differences in psychological distress [*F*(2, 8,112) = 545.973, *p* < 0.001, η^2^ = 0.119], suicidal ideation [*F*(2, 8,112) = 318.600, *p* < 0.001, η^2^ = 0.073], family self-concept [*F*(2, 8,112) = 251.839, *p* < 0.001, η^2^ = 0.058], and social self-concept [*F*(2, 8,111) = 32.288, *p* < 0.001, η^2^ = 0.008] (see Table 3). The results obtained in the Bonferroni test (α = 0.05) showed that adolescents with high CPV obtained the highest scores in psychological distress, and suicidal ideation, whereas those with low CPV obtained the highest scores in family self-concept and social self-concept. The effect size of η^2^p is low and moderate (between 0.008 and 0.119).

**TABLE 3:** Means, standard deviation (SD), and ANOVA results of CPV and suicidal ideation, psychological distress, family self-concept, and social self-concept.

	CPV			*F*	η^2^
	Low	Moderate	High	*F*(2, 8,112)	
SI	1.378 (0.586)^c^	1.775 (0.818)^b^	2.130 (0.893)^a^	318.600***	0.073^††^
PD	1.893 (0.765)^b^	2.622 (0.930)^a^	2.752 (1.004)^a^	545.973***	0.119^††^
FSC	82.079 (18.198)^a^	71.967 (22.728)^b^	60.526 (22.010)^c^	251.839***	0.058^†^
SSC	76.349 (18.012)^ a^	76.410 (18.322)^a^	65.208 (22.040)^b^	32.288***	0.008

SI, suicidal ideation; PD, psychological distress; FSC, family self-concept; SSC, social self-concept. Bonferroni test α = 0.05, a > b > c; ***p < 0.001; η^2^ = 0.01-0.06 (small effect †), > 0.06-0.14 (moderate effect ††).

Regarding gender, the ANOVA results showed significant differences in psychological distress [*F*(1, 8,113) = 572.304, *p* < 0.001, η^2^ = 0.066], suicidal ideation [*F*(1, 8,113) = 197.974, *p* < 0.001, η^2^ = 0.024], and family self-concept [*F*(1, 8,113) = 18.774, *p* < 0.001, η^2^ = 0.002]. As shown in Table 4, girls scored higher than boys in psychological distress and suicidal ideation, while boys scored higher in family self-concept.

**TABLE 4:** Means, standard deviation (SD), and ANOVA results of gender, suicidal ideation, psychological distress, family self-concept, and social self-concept.

	Gender		*F*	η^2^
	Boys	Girls	*F*(1, 8,113)	
SI	1.363 (0.564)	1.568 (0.743)	197.974***	0.024^†^
PD	1.824 (0.738)	2.263 (0.909)	572.304***	0.066^††^
FSC	80.797 (18.334)	78.901 (21.052)	18.774***	0.002
SSC	75.915 (17.827)	76.335 (18.650)	1.075	0.000

SI, suicidal ideation; PD, psychological distress; FSC, family self-concept; SSC, social self-concept. ***p < 0.001.

### Univariate Analyses of Interaction Effects

**FIGURE 1:**
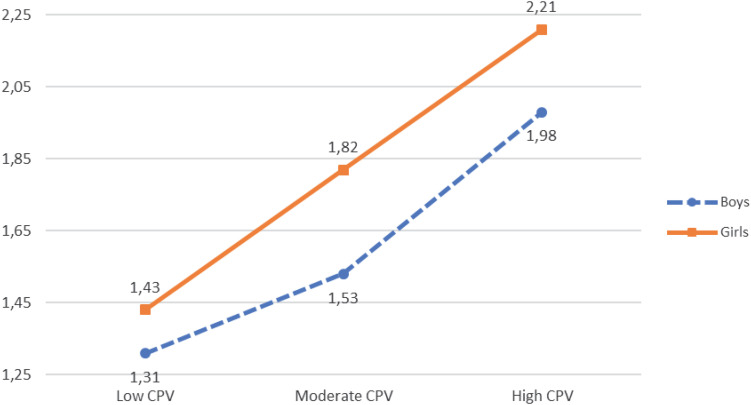
CPV, gender, and suicidal ideation.

A statistically significant interaction effect was obtained between CPV, gender, and psychological distress [*F*(2, 8,108) = 17.049, *p* < 0.001, η^2^ = 0.004]. The results of the *post hoc* contrasts performed with the Bonferroni test (α = 0.05) (see Table 5 and Figure 1) indicated that when CPV was low, moderate, or high, girls reported higher scores in psychological distress than boys. However, when CPV was high, girls were the ones with the highest psychological distress.

**TABLE 5:** Means, standard deviation (SD) between CPV and gender and suicidal ideation, psychological distress, and family self-concept.

	CPV and gender						*F*	η^2^
	Low CPV		Moderate CPV		High CPV			
	Boys	Girls	Boys	Girls	Boys	Girls	*F*(2, 8,108)	
SI	1.31^f^ (0.514)	1.43^e^ (0.636)	1.53^d^ (0.641)	1.82^c^ (0.836)	1.98^b^ (0.849)	2.21^a^ (0.944)	14.311***	0.004
PD	1.72^e^ (0.671)	2.04^d^ (0.804)	2.21^c^ (0.799)	2.74^b^ (0.895)	2.67^b^ (0.933)	3.16^a^ (0.916)	17.049***	0.004
FSC	82.46^a^ (17.445)	82.52^a^ (18.441)	75.55^b^ (19.534)	71.95^c^ (23.430)	62.99^d^ (21.097)	61.81^e^ (25.703)	9.396***	0.002

SI, suicidal ideation; PD, psychological distress; FSC, family self-concept. ***p < 0.001. a > b > c > d > e > f.

**FIGURE 2:**
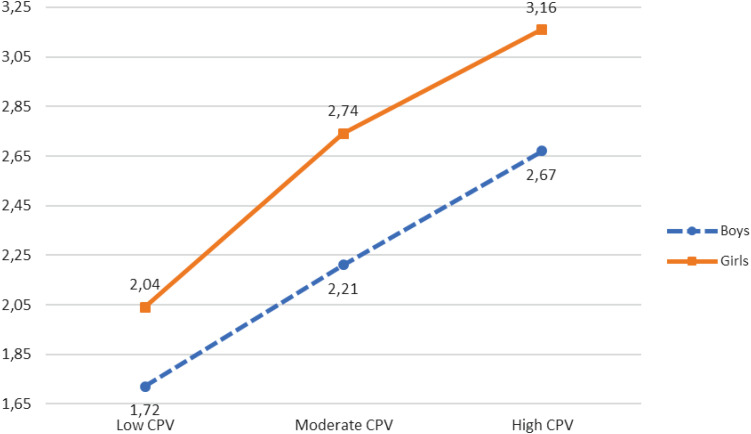
CPV, gender, and psychological distress.

Thus, a statistically significant interaction effect was observed between CPV, gender, and suicidal ideation [*F*(2, 8,108) = 14.311, *p* < 0.001, η^2^ = 0.004]. The results of the *post hoc* contrasts performed with the Bonferroni test (α = 0.05) (see Table 5 and Figure 2) revealed that when CPV was low, moderate, or high, boys obtained lower scores in suicidal ideation than girls. In addition, when CPV was high, girls scored higher than boys in suicidal ideation.

**FIGURE 3:**
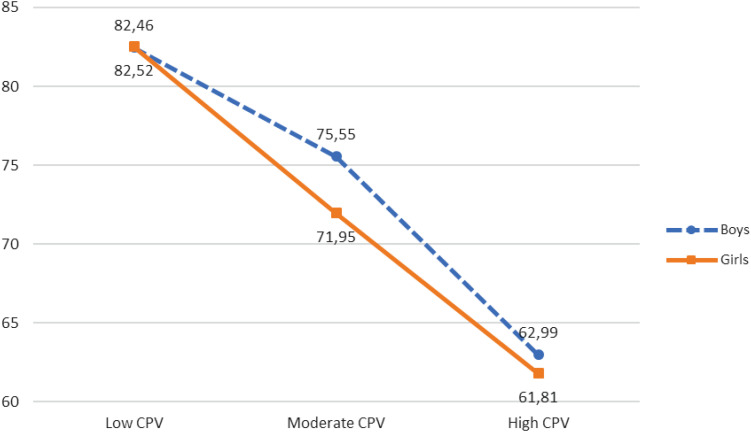
CPV, gender, and family self-concept.

Further, a statistically significant interaction effect between CPV, gender, and family self-concept [*F*(2, 8,108) = 9.396, *p* < 0.001, η^2^ = 0.002] was also obtained. As illustrated in Table 5 and Figure 3, when CPV was low or high, no statistically significant differences were found between girls and boys, However, in moderate CPV, boys showed higher scores in family self-concept than girls.

## Discussion

This study aimed to broaden knowledge of CPV and its association with various maladjustment outcomes. Based on the General Strain Theory, this research examined the relationships between CPV, psychological distress, suicidal ideation, and family and social self-concept, according to the gender of adolescents. Firstly, results indicated that CPV levels increased as psychological distress increased, confirming our hypothesis. These findings are consistent with those found in previous studies highlighting the direct association between adolescent aggression toward parents and outcomes of psychological distress such as depressive symptoms and anxiety (Kennedy et al., 2010; Gámez-Guadix et al., 2012; Sanchez-Meca et al., 2016). Our results add to a body of research showing that adolescents engaged in CPV are likely to suffer from stress, and suggests that CPV is also associated with higher levels of psychological distress, thus supporting the General Strain Theory (Brezina, 1999; Agnew, 2006). This theory posits that CPV could be a strategy to respond to previous aversive family interactions and reduce stress (Ibabe, 2019).

Secondly, regarding suicidal ideation, the findings are very similar to the ones above. As expected, our findings showed that greater involvement in CPV resulted in higher levels of suicidal ideation. Previous studies have reported the relationship between suicidal ideation and other forms of violence, such as bullying and cyberbullying (Hinduja and Patchin, 2010; Litwiller and Brausch, 2013; Barzilay et al., 2017); however, few studies have analyzed its link with CPV. Our results suggests that CPV could be the expression of core maladjustment outcomes in adolescents such as suicidal ideation, which is in turn associated with psychological distress. Moreover, psychological distress, depression, anxiety, and suicidal ideation are likely to co-occur in adolescence (Juon et al., 1994; O’Leary et al., 2006; Sampasa-Kanyinga and Hamilton, 2016; Iranzo et al., 2019) indicating that these adolescents cope with a set of adverse experiences in a negative way. Previous research has highlighted that negative family relationships and exposure to family violence, and hazardous and negative parent–child relationships increase adolescent psychological distress, which is in turn related to CPV (Beckmann, 2019; Calvete et al., 2020). Similarly, Contreras et al. (2016) found that adolescents who abused their parents reported higher levels of exposure to violence not only at home but also in the school and community. Future research should further explore the links between negative family relationships, psychological distress, and suicidal ideation on the one hand, and CPV on the other.

Thirdly, with regard to the second hypothesis, the results showed that CPV levels increased as family and social self-concept decreased. These results are consistent with those reported in previous studies for other forms of violence, and also they add scientific evidence to support the fact that adolescents who engage in CPV have psychological adjustment problems (Forrester et al., 2008; Calvete et al., 2014; Ibabe and Bentler, 2016; Sanchez-Meca et al., 2016). More specifically, teens with CPV problems have few resources to face stressful and adverse experiences, such as self-concept (Calvete et al., 2011), in cases of CPV. It is important to underline that most studies have used unidimensional and general measures of self-concept or self-esteem. This result provides interesting new data on CPV in adolescents by examining two specific dimensions of self-concept. Interestingly, our study showed that both family and social self-concept are important resources associated with lower levels of CPV. This result diverges from the findings reported in previous studies examining other forms of violence such as bullying, which found that perpetrators reported higher levels of social self-esteem and self-concept (Estévez et al., 2006; Fuentes et al., 2011). Our findings suggest that CPV is particularly rooted in adverse family dynamics. This result can be considered highly significant and these relationships should be analyzed in greater depth in future research.

Finally, in terms of gender differences, the results showed that girls reported higher levels of psychological distress and suicide ideation and lower levels of family self-concept than boys. However, outcomes for the interaction between CPV and gender revealed the need for more in-depth analysis of these relationships. Findings obtained indicated that girls scored higher on psychological distress than boys and as their involvement in CPV was more frequent, the differences between boys and girls were greater. This trend was observed for suicidal ideation too. Girls showed more suicidal ideation than boys, especially those with high CPV. These results are in line with prior studies (Hinduja and Patchin, 2010; Nock et al., 2013) and with those that have analyzed gender differences in other forms of violence (Romero-Abrio et al., 2018). Specific studies on CPV have also found that girls with psychopathological problems (anxiety, depression, and paranoid ideation, among others) exhibit greater CPV than boys (Rosado et al., 2017). However, our results differ from those reported in other studies in which no differences were found between boys and girls (Williams et al., 2017). This result can be attributed to the fact that, on the one hand, girls tend to report higher levels of depression, anxiety, and stress than boys (Hamilton et al., 2016; Hébert et al., 2016) and, on the other, girls are more sensitive to problems in the family, an aspect that is closely related to CPV. In this sense, in this study girls with high CPV reported the lowest scores in family self-concept; hence, these adolescents feel less valued and less accepted by their parents. These findings highlight the role of family relationships in CPV and psychological distress. However, more research is needed to explore the effect of family self-concept on the relationship between CPV and psychological distress taking into account gender differences.

## Limitations

First, variables did not exhibit causal relationships because a cross-sectional design was applied. Longitudinal studies should therefore be carried out in the future. Second, these findings were obtained from a Mexican sample and could influence the generalization of the study results. Further research in other countries is needed to address these findings in other cultural contexts. In addition, this research was conducted with a school sample, thus, in future studies it would be interesting to expand the sample in other areas of study, such as in clinical and judicial samples. Moreover, it is important to highlight that this study was carried out with self-report measures, therefore, obtaining information from fathers, mothers, and teachers is necessary in order to better understand the relationships between the variables analyzed. Likewise, these results should be interpreted with caution because the Conflict Tactics Scale (CTS2) does not have a time parameter and could lead to recall bias due to the difficulty in accurately and completely remembering previous memories. Future research that includes the temporal dimension would allow us to further develop the findings obtained. Finally, we mentioned the differences found between CPV toward mothers and CPV toward fathers in previous research at the introduction of this work. However, these differences have not been analyzed in our study, and we consider it worthwhile to separately examine these dimensions of CPV, in order to advance the understanding of this kind of violence.

## Conclusion

The findings obtained in this study provide, in our opinion, relevant information in the field of psychology and education regarding the relationships between CPV and psychological distress, suicidal ideation, and self-concept in school-aged adolescents. It has been demonstrated that these three variables are significantly related, requiring an expansion of knowledge in the field of psychology and education. Thus far, these variables as a whole have been little explored. Findings reveal that CPV perpetrators, especially girls, also show maladjustment problems such as psychological distress, suicidal ideation, and poor family self-concept. Although boys are more frequently involved in CPV, girls show greater maladjustment problems. Our results also suggest the importance of examining self-concept from a multidimensional perspective. These findings underline that different self-concept domains are more related to different forms of violence. While social self-concept is important when examining peer aggression, family self-concept has been found to be especially relevant for understanding CPV. Finally, based on these findings, intervention programs should take into account that girls engaging in CPV also have more maladjustment outcomes. For example, actions should be implemented to foster family self-concept and to help adolescents involved in CPV (especially girls) and their families cope with stressful situations in the family. In addition, some relevant implications for prevention and intervention programs in CPV were identified. On the one hand, the appropriateness of continuing to develop educational and treatment programs that promote networking and jointly consider different areas of intervention: school, family, and individual, should be considered; on the other hand, the need to consider psychological distress, suicidal ideation, and self-concept in the design of CPV prevention and intervention programs is highlighted.

## Data Availability Statement

The raw data supporting the conclusions of this article will be made available by the authors, without undue reservation, to any qualified researcher.

## Ethics Statement

The studies involving human participants were reviewed and approved by the Institutional Review Board of the Faculty of Psychology of Autonomous University of Nuevo Leon. Written informed consent to participate in this study was provided by the participants’ legal guardian/next of kin.

## Author Contributions

All authors of the manuscript contributed equally to the research and writing of the present study.

## Conflict of Interest

The authors declare that the research was conducted in the absence of any commercial or financial relationships that could be construed as a potential conflict of interest.
